# Psychological well-being trajectories preceding incident mild cognitive impairment and dementia

**DOI:** 10.1136/jnnp-2024-333837

**Published:** 2024-08-13

**Authors:** Jie Guo, Jiao Wang, Abigail Dove, David A Bennett, Weili Xu

**Affiliations:** 1 Department of Nutrition and Health, China Agricultural University, Beijing, China; 2 Aging Research Center, Department of Neurobiology, Care Sciences and Society, Karolinska Institutet, Stockholm, Sweden; 3 Department of Epidemiology, College of Preventive Medicine, Army Medical University, Chongqing, China; 4 Rush Alzheimer's Disease Center, Rush University Medical Center, Chicago, Illinois, USA

**Keywords:** DEMENTIA

## Abstract

**Background:**

Poorer psychological well-being has been related to an increased dementia risk, but changes in psychological well-being along the dementia course are unclear. We explored psychological well-being trajectories before and after the diagnosis of mild cognitive impairment (MCI) and dementia.

**Methods:**

Within the Rush Memory and Aging Project, 910 cognitively intact older adults were followed annually for up to 14 years to detect incident MCI and dementia. Psychological well-being and its six components (self-acceptance, autonomy, environmental mastery, purpose in life, positive relation with others, and personal growth) were annually measured based on Ryff’s Scales of Psychological Well-Being. Data were analysed using mixed-effect models with a backward timescale.

**Results:**

Compared with participants who remained cognitively intact, those who developed incident MCI had a faster decline in psychological well-being (β −0.015, 95% CI −0.027 to –0.003), leading to lower well-being 2 years before MCI diagnosis (mean difference at year −2, −0.099, 95% CI −0.187 to –0.012). Considering different well-being components, those who developed MCI had lower levels of purpose in life and personal growth beginning 3 years (−0.126, 95% CI −0.251 to –0.001) and 6 years (−0.139, 95% CI −0.268 to –0.009) before MCI, respectively. The slope of psychological well-being decline was similar before and after MCI diagnosis for each component except for positive relation with others, which had an accelerated decline after MCI (β −0.042, 95% CI−0.075 to –0.009). Well-being trajectories remained similar for individuals with MCI regardless of whether they later developed dementia.

**Conclusions:**

Psychological well-being (specifically purpose in life and personal growth) became significantly lower before MCI diagnosis.

**WHAT IS ALREADY KNOWN ON THIS TOPIC**

- Psychological well-being has been linked with dementia, whereas the trajectories of psychological well-being and its components along the dementia course are unclear.

**WHAT THIS STUDY ADDS**

- Psychological well-being, especially the terms purpose in life and personal growth, became significantly lower preceding MCI diagnosis. Positive relations with others declined faster after MCI diagnosis than that before. Among MCI participants, well-being declined in similar patterns regardless of whether developing dementia or not.

**HOW THIS STUDY MIGHT AFFECT RESEARCH, PRACTICE OR POLICY**

- Reduced psychological well-being, even in the absence of evident cognitive impairment, may serve as a predictor for impaired cognitive function, and post-diagnostic psychological support should be planned for people diagnosed with dementing disorders.

## Introduction

 Dementia is a global concern. Worldwide, there were an estimated 57.4 million dementia cases, and this is projected to triple by 2050.^1^ Dementia has been associated with a wide range of adverse health consequences, including disability and mortality, and imposes substantial burdens on families and societies.^2^ While dementia has no curative treatment, dementia’s long preclinical stage may provide a critical time window for implementing preventions to prevent or delay its onset. Therefore, identifying populations at high-risk of dementia earlier is crucial to implement preventive measures and reduce dementia incidence and associated burdens.

Accumulating evidence has linked psychological well-being to cognitive ageing.^3^ There may be bidirectional associations between cognitive function and psychological well-being.^4^ On the one hand, several longitudinal studies have shown that lower levels of psychological well-being were associated with increased risk of cognitive impairment and dementia among older adults.^5^,^8^ A recent meta-analysis combined eight samples and found that a greater sense of meaning/purpose in life—one facet of well-being—was associated with lower dementia risk.^9^ Consistent with this, our prior research from the Rush Memory and Aging Project (MAP) also showed that having a greater purpose in life was linked to lower risk of mild cognitive impairment (MCI) and dementia,^5^ and may extend dementia-free survival.^10^ Moreover, a higher level of psychological well-being may mitigate the adverse effects of Alzheimer’s disease pathology on cognition.^11^ On the other hand, rapid cognitive decline and dementia have been linked to lower levels of psychological well-being.^12^ Individuals diagnosed with dementia often have difficulties in adapting adequately to situation changes, establishing new objectives, and maintaining previous relationships with others, which could influence their psychological well-being.^13^ To date, only one study has assessed changes in purpose in life before and after the development of cognitive impairment, showing that purpose in life declined significantly before the onset of cognitive impairment and declined at a much faster rate thereafter.^14^ Understanding how psychological well-being changes throughout the progression of dementing disorders could better inform its potential role as a predictor of dementia risk and provide relevant information for healthcare planning after diagnosis. Moreover, psychological well-being is not a unitary construct, and it is possible to hypothesise that different aspects of psychological well-being may have different trajectories during the progression of dementing disorders. However, most previous studies have focused on only one facet—meaning/purpose in life.^5 6 9 10 14^ Evidence for other components of psychological well-being besides purpose in life is needed.

In this study, using longitudinal data from the Rush MAP, we aim to build on previous research by exploring the trajectories of psychological well-being and its different components before and after MCI and dementia diagnoses.

## Methods

### Study design and participants

MAP is an ongoing longitudinal cohort study that began in 1997 and includes older adults recruited from various sources, including senior and subsidised housing, continuous care retirement communities, social service agencies, church groups, and individual homes in northeastern Illinois.^15^ Participants undergo annual follow-up assessments that include neurological examination, cognitive tests, medical history, and psychological well-being.^15^

Here, we considered 2008 as the analytical baseline since it was the year when annual data on psychological well-being was augmented from just purpose in life to an 18-item multidomain assessment. Participants were annually followed for up to 14 years. A total of 910 participants who were cognitively intact (ie, free of MCI and dementia) at baseline were included in the analysis of well-being trajectory before and after incident MCI. Of them, 265 had incident MCI over the follow-up. After excluding 36 participants without any measurements of well-being after MCI diagnosis, we included 229 participants for the analysis of well-being trajectory before and after incident dementia (online supplemental figure 1).

This study was approved by an Institutional Review Board of Rush University Medical Center and adhered to the ethical standards in the 1964 Declaration of Helsinki and its subsequent amendments. Before enrolment, all individuals provided informed consent. Moreover, participants signed a repository consent form, granting permission for their data to be shared.

### Assessment of psychological well-being

The 18-item version of Ryff’s Scales of Psychological Well Being was used to assess the well-being at baseline and thereafter.^11 16^ The scale includes three items on each of six well-being components: self-acceptance, autonomy, environmental mastery, purpose in life, positive relations with others, and personal growth (online supplemental table 1). Each item is scored on a 7-point Likert scale. The total score is the average of the ratings, with a higher score indicating greater well-being (range 1 to 7).

### Assessment of MCI and dementia

Clinical diagnoses of dementia followed a standardised and structured process, incorporating computerised cognitive test scoring, clinical evaluation by a neuropsychologist who determined the presence of cognitive impairment, and diagnostic classification of dementia by a clinician.^17 18^ The diagnostic criteria used for dementia was based on the joint working group of the National Institute of Neurological and Communicative Disorders and Stroke and the Alzheimer’s Disease and Related Disorders Association.^17^ A diagnosis of MCI was rendered for participants who were judged by a neuropsychologist to have objective impairment but did not receive a diagnosis of dementia from the examining clinician.^18 19^

### Covariates

Information on sociodemographic factors (ie, age, sex, and education) and lifestyle factors was collected at study entry. Education was recorded as the duration of formal schooling in years. Physical activity was expressed as the total number of hours per week that participants engaged in from five types of activities (ie, gardening or yard work, walking for exercise, bicycle riding, callisthenics or general exercise, and swimming or water exercise) within the past 2 weeks using questions adapted from the National Health Interview Survey.^20^ Body mass index (BMI) was calculated using weight (kg) divided by height squared (m^2^). Vascular disease risk factors included hypertension, diabetes, and smoking history. Vascular diseases, including stroke, claudication, heart conditions (ie, heart attack or coronary, coronary occlusion, or coronary thrombosis), and congestive heart failure, were self-reported by participants. Stroke was also ascertained based on clinical and neurological examination, and cognitive testing. Depressive symptoms were assessed with a modified 10-item version of the Center for Epidemiologic Studies Depression Scale.^21^ Social activity was assessed based on participants’ self-reported level of engagement in six items during the past year (go to restaurants, sporting events or teletract, or play bingo; day trips or overnight trips; unpaid community or volunteer work; visit at relatives’ or friends’ houses; participant in groups; attend church or religious services). Social network refers to the number of individuals (children, family, and friends) that participants interact with at least once a month. Loneliness refers to perceived social isolation and feeling disconnected from others and was assessed using five items from a modified version of the de Jong-Gierveld Loneliness Scale.

Apolipoprotein E (*APOE*) alleles were assessed from blood samples. Participants were dichotomised as *APOE* ε4 carriers versus non-carriers.

### Statistical analyses

Baseline characteristics of the study population by incident MCI status were compared using χ^2^ tests for categorical variables and t-test for continuous variables.

To examine the trajectories of psychological well-being before and after MCI, a backward and forward timescale was used, where the year 0 corresponded to the year of MCI diagnosis for participants who developed MCI and to the end of follow-up for those who remained cognitively intact. Moreover, among participants with incident MCI, we included a change point in relation to the year 0 (ie, the year of MCI diagnosis). Piecewise linear mixed-effect models were used to explore well-being trajectories, with cognitive status, time, and their interaction included in the model to test the differences in well-being trajectories by cognitive status (reference: cognitively intact). Detailed information about the model is provided in online supplemental text 1. The random effect included a random intercept for individuals at year 0 and a slope for time, assuming an unstructured covariance structure. The difference in well-being between cognitively intact participants and those who developed MCI was estimated for each year preceding year 0, with a negative value indicating lower well-being among the MCI group. We further compared the slope of well-being changes between participants who developed into MCI and those who kept cognitively intact, and before and after MCI diagnosis among those with incident MCI. All analyses were adjusted for age at time 0, sex, and education. We also included the interaction terms between these variables and time when the value for interactions was <0.05. Similarly, among participants with incident MCI, we analysed trajectories before and after dementia diagnosis, using the year of incident dementia or the last follow-up as year 0 (reference: non-dementia during the follow-up).

In supplementary analyses, we conducted multi-adjusted models with adjustment for age at time 0, sex, education, physical activity, BMI, vascular risk factors (ie, smoking, hypertension, and diabetes), vascular diseases (ie, claudication, stroke, and heart conditions), depression syndrome, *APOE* ε4 carrier status, social activity, social network, and loneliness. Statistical analyses were performed using SAS 9.4 (SAS Institute, Cary, NC, USA). All p values were two-sided, and we defined statistical significance as p<0.05. In analyses comparing the estimated mean well-being at various time-points preceding diagnosis between individuals with and without MCI, as well as between those with dementia and those who were dementia-free, we used a simulation-based approach combined with a step-down fashion to calculate adjusted p values and confidence intervals (95% CI). This method can account for the multiple testing.^22^

## Results

### Baseline characteristics

A total of 910 participants were included in the analysis. At baseline, mean (SD) age was 79.9 (7.5) years and 76.9% were female. During the follow-up (median (IQR) duration 6 (3–10) years), 265 (29.1%) participants developed MCI. Of them, 89 (33.6%) developed dementia. Compared with participants who remained cognitively intact, those who developed MCI were more likely to be older, have lower BMI, and have lower levels of depressive symptoms and psychological well-being (p<0.05 for all) (table 1). Compared with participants who were dementia-free, those who developed dementia were more likely to be older, female, *APOE* ε4 carriers, and to have a lower level of psychological well-being (p<0.05 for all).

**Table 1:** Baseline characteristics of the study population by incident cognitive impairment in the cognitively intact group and by incident dementia in the incident group

Characteristics	Total(n=910)	Among cognitively intact participants			Among incident MCI participants		
		MCI-free(n=645)	Incident MCI(n=265)	P value	Dementia-free(n=156)	Incident dementia(n=73)	P value
Age at baseline (years), mean (SD)	79.9 (7.5)	78.9 (7.7)	82.1 (6.5)	<0.001	81.3 (6.4)	83.8 (6.0)	0.005
Sex, n (%)				0.126			0.035
Female	700 (76.9)	505 (78.3)	195 (73.6)		105 (67.3)	59 (80.8)	
Male	210 (23.1)	140 (21.7)	70 (26.4)		51 (32.7)	14 (19.2)	
Education (years), mean (SD)	15.6 (3.1)	15.7 (3.1)	15.6 (3.2)	0.859	15.7 (3.0)	15.0 (3.1)	0.117
Physical activity (hours/week), mean (SD)	3.8 (3.7)	3.7 (3.7)	4.0 (3.8)	0.290	4.2 (4.2)	3.4 (3.1)	0.098
Body mass index (kg/m^2^), mean (SD)	27.6 (5.6)	28.1 (5.9)	26.4 (4.7)	<0.001	26.6 (4.8)	26.2 (4.6)	0.629
Any of vascular disease risk factors*, n (%)	704 (77.3)	496 (76.9)	208 (78.5)	0.613	128 (82.1)	58 (79.5)	0.913
Any vascular diseases†, n (%)	212 (23.3)	148 (22.9)	64 (24.1)	0.443	38 (24.4)	20 (27.4)	0.689
Depression syndrome, mean (SD)	0.9 (1.4)	0.9 (1.5)	0.7 (1.3)	0.011	0.7 (1.2)	0.7 (1.4)	0.899
Apolipoprotein E ε4 carriers, n (%)	136 (14.9)	79 (12.2)	57 (21.5)	0.079	27 (17.3)	23 (31.5)	0.042
Social activity score, mean (SD)	2.7 (0.6)	2.7 (0.6)	2.7 (0.5)	0.504	2.8 (0.5)	2.7 (0.5)	0.128
Social network size, mean (SD)	7.5 (6.1)	7.4 (6.1)	7.7 (5.9)	0.535	7.5 (5.7)	8.4 (6.9)	0.347
Loneliness score, mean (SD)	2.1 (0.6)	2.1 (0.6)	2.1 (0.6)	0.126	2.2 (0.6)	2.1 (0.6)	0.636
Psychological well-being, mean (SD)	5.7 (0.6)	5.7 (0.6)	5.6 (0.5)	0.015	5.6 (0.5)	5.4 (0.6)	0.011

* Vascular disease risk factors included smoking, hypertension, and diabetes.

† Vascular diseases included claudication, stroke, heart conditions (ie, heart attack or coronary, coronary thrombosis, coronary occlusion, or myocardial infarction), and congestive heart failure.

MCI, mild cognitive impairment.

### Trajectories of psychological well-being before and after MCI diagnosis

**Figure 1:**
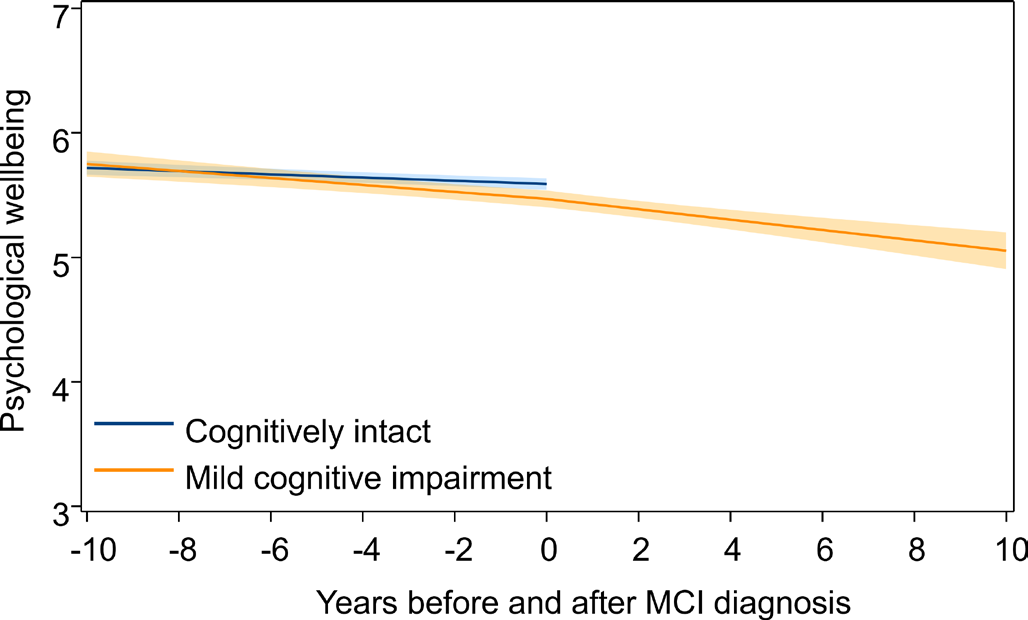
Trajectories of psychological well-being before and after mild cognitive impairment (MCI) diagnosis. Model was adjusted for age at time 0, sex, and education. Year 0 indicates the year of MCI diagnosis or the year corresponding to the end of follow-up (for participants who remained cognitively intact). Psychological well-being declined faster in cognitively intact participants (β coefficient −0.013, 95% CI –0.019 to –0.007, p<0.001) and MCI participants (β coefficient −0.028, 95% CI −0.038 to –0.018, p<0.001). Difference between β coefficients of cognitively intact participants and MCI participants before diagnosis was −0.015 (95% CI –0.027 to –0.003) (p=0.014). After the diagnosis, psychological well-being declined faster (−0.042, 95% CI –0.056 to 0.027, p<0.001). Difference between β coefficients of MCI participants before and after diagnosis was −0.014 (95% CI –0.034 to 0.007) (p=0.187).

Figure 1 shows the trajectories of psychological well-being before and after MCI diagnosis. Psychological well-being declined faster among participants with incident MCI compared with those who remained cognitively intact (β coefficient of MCI vs cognitively intact: −0.015, 95% CI −0.027 to –0.003, p=0.014), leading to a lower level of well-being starting from 2 years before MCI diagnosis (difference in mean at year −2: −0.099, 95% CI −0.187 to –0.012) (table 2). After the MCI diagnosis, well-being tended to decline faster, though the difference in slope before and after diagnosis was not statistically significant (−0.014, 95% CI −0.034 to 0.007, p=0.187). In the multi-adjusted model, psychological well-being tended to decline faster among participants with incident MCI compared with those who remained cognitively intact (β coefficient of MCI vs cognitively intact: −0.012, 95% CI −0.024 to 0.000, p=0.058) (online supplemental table 2).

**Table 2:** Differences in psychological well-being between MCI cases and those cognitively intact in the 10 years before MCI diagnosis

Year	Number of cognitively intact participants	Number of MCI	Difference in mean(95% CI)	P value
−10	189	40	0.023 (−0.112 to 0.158)	0.745
−9	230	51	0.008 (−0.117 to 0.132)	0.905
−8	262	64	−0.008 (−0.123 to 0.107)	0.905
−7	297	87	−0.023 (−0.129 to 0.083)	0.693
−6	342	115	−0.038 (−0.137 to 0.061)	0.456
−5	382	130	−0.054 (−0.147 to 0.040)	0.255
−4	464	157	−0.069 (−0.158 to 0.020)	0.119
−3	499	184	−0.084 (−0.171 to 0.003)	0.050
−2	500	205	**−0.099 (−0.187 to −0.012**)	**0.021**
−1	548	251	**−0.115 (−0.205 to −0.024**)	**0.009**
0	645	265	**−0.130 (−0.225 to −0.035**)	**0.005**

Difference in mean was calculated as the mean of well-being in participants with MCI minus that in those cognitively intact. Negative value means that well-being was poorer in participants with MCI. Model was adjusted for age at time 0, sex, and education.

Year 0 indicates the year of MCI diagnosis or the year corresponding to the end of follow-up (for participants who remained cognitively intact).

To account for the multiple testing, Pp- values and confidence intervals (95% CI) in the table were calculated using a simulation-based approach combined with a step-down fashion.

MCI, mild cognitive impairment.

Of six components of well-being, autonomy declined faster among participants with incident MCI compared with those who remained cognitively intact (β coefficient of MCI vs cognitively intact: −0.021, 95% CI –0.04 to –0.003, p=0.020) (online supplemental figure 2 and online supplemental table 3), leading to a lower level of autonomy at MCI diagnosis (difference in mean at year 0: −0.139, 95% CI –0.266 to –0.012) (table 3). Environmental mastery, purpose in life, positive relation with others, and personal growth tended to decline faster before MCI diagnosis, although not significantly (online supplemental figure 2 and online supplemental table 3). Compared with participants who remained cognitively intact, those who developed MCI had a lower level of purpose in life beginning 3 years before MCI diagnosis (difference in mean at year −3: −0.126, 95% CI –0.251 to –0.001) and a lower level of personal growth beginning 6 years before MCI diagnosis (difference in mean at year −6: −0.139, 95% CI –0.268 to –0.009) (table 3). Among participants with MCI, positive relation with others declined faster after than before MCI diagnosis (−0.042, 95% CI –0.075 to –0.009, p=0.012) (online supplemental table 3).

**Table 3:** Differences in components of psychological well-being between MCI cases and those cognitively intact in the 10 years before MCI diagnosis

Year	Self-acceptance	Autonomy	Environmental mastery	Purpose in life	Positive relation with others	Personal growth
	Difference in mean (95% CI)	Difference in mean (95% CI)	Difference in mean (95% CI)	Difference in mean (95% CI)	Difference in mean (95% CI)	Difference in mean (95% CI)
−10	−0.027 (−0.236 to 0.182)	0.076 (−0.129 to 0.281)	0.077 (−0.148 to 0.303)	−0.017 (−0.234 to 0.199)	0.107 (−0.122 to 0.337)	−0.114 (−0.305 to 0.077)
−9	−0.032 (−0.225 to 0.160)	0.054 (−0.133 to 0.242)	0.060 (−0.145 to 0.264)	−0.033 (−0.230 to 0.164)	0.094 (−0.118 to 0.305)	−0.120 (−0.294 to 0.054)
−8	−0.038 (−0.214 to 0.139)	0.033 (−0.139 to 0.204)	0.042 (−0.142 to 0.226)	−0.048 (−0.227 to 0.130)	0.080 (−0.114 to 0.274)	−0.126 (−0.284 to 0.031)
−7	−0.043 (−0.205 to 0.120)	0.011 (−0.145 to 0.168)	0.024 (−0.141 to 0.189)	−0.064 (−0.226 to 0.098)	0.066 (−0.112 to 0.244)	−0.132 (−0.275 to 0.010)
−6	−0.048 (−0.198 to 0.102)	−0.010 (−0.153 to 0.133)	0.006 (−0.142 to 0.155)	−0.079 (−0.227 to 0.069)	0.053 (−0.111 to 0.216)	**−0.139 (−0.268 to −0.009**)
−5	−0.053 (−0.193 to 0.087)	−0.032 (−0.164 to 0.101)	−0.012 (−0.146 to 0.123)	−0.095 (−0.231 to 0.042)	0.039 (−0.112 to 0.190)	**−0.145 (−0.263 to −0.026**)
−4	−0.058 (−0.191 to 0.075)	−0.053 (−0.177 to 0.071)	−0.029 (−0.155 to 0.096)	−0.110 (−0.239 to 0.018)	0.025 (−0.116 to 0.166)	**−0.151 (−0.261 to −0.040**)
−3	−0.063 (−0.193 to 0.066)	−0.075 (−0.194 to 0.045)	−0.047 (−0.168 to 0.074)	**−0.126 (−0.251 to −0.001**)	0.011 (−0.123 to 0.146)	**−0.157 (−0.263 to −0.050**)
−2	−0.068 (−0.198 to 0.061)	−0.096 (−0.214 to 0.022)	−0.065 (−0.186 to 0.057)	**−0.141 (−0.268 to −0.015**)	−0.002 (−0.134 to 0.129)	**−0.163 (−0.270 to −0.056**)
−1	−0.074 (−0.207 to 0.060)	−0.117 (−0.238 to 0.003)	−0.083 (−0.211 to 0.045)	**−0.157 (−0.289 to −0.024**)	−0.016 (−0.149 to 0.117)	**−0.169 (−0.280 to −0.058**)
0	−0.079 (−0.219 to 0.062)	**−0.139 (−0.266 to −0.012**)	−0.100 (−0.239 to 0.038)	**−0.172 (−0.314 to −0.030**)	−0.030 (−0.168 to 0.108)	**−0.175 (−0.294 to −0.056**)

Difference in mean was calculated as the mean of corresponding well-being in participants with MCI minus that in those cognitively intact. Negative value means that corresponding well-being was poorer in participants with MCI. Model was adjusted for age at time 0, sex, and education.

Year 0 indicates the year of MCI diagnosis or the year corresponding to the end of follow-up (for participants who remained cognitively intact).

To account for the multiple testing, -p values and confidence intervals (95% CI) in the table were calculated using a simulation-based approach combined with a step-down fashion.

MCI, mild cognitive impairment.

### Trajectories of psychological well-being before and after dementia diagnosis

**Figure 2:**
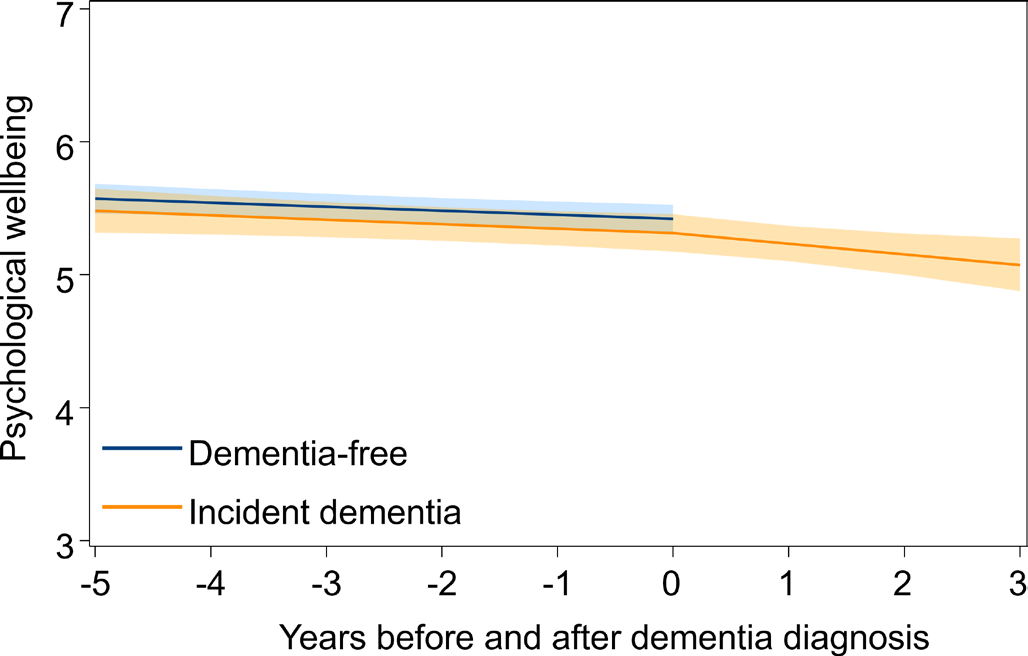
Trajectories of psychological well-being before and after dementia diagnosis among participants with incident mild cognitive impairment. Model was adjusted for age at time 0, sex, and education. Year 0 indicates the year of dementia diagnosis or the year corresponding to the end of follow-up (for participants who were dementia-free). Psychological well-being declined faster in dementia-free participants (β coefficient −0.030, 95% CI –0.052 to –0.009*,* p=0.006) and dementia participants (−0.033, 95% CI –0.067 to 0.000, p=0.053). Difference between β coefficients of dementia-free participants and dementia participants before diagnosis was −0.003 (95% CI –0.043 to 0.037) (p=0.875). After the diagnosis, psychological well-being declined faster (−0.080, 95% CI –0.146 to –0.014, p=0.018). Difference between β coefficients of dementia cases before and after diagnosis was −0.046 (95% CI –0.134 to 0.041) (p=0.289).

Figure 2 shows the trajectories of psychological well-being before and after dementia diagnosis among participants with incident MCI. The trajectories of psychological well-being were similar regardless of future dementia development (β coefficient of dementia vs dementia-free: −0.003, 95% CI −0.043 to 0.037, p=0.875). There was no statistically significant difference in mean well-being preceding dementia diagnosis (online supplemental table 4). Moreover, the decline in psychological well-being after dementia diagnosis tended to be faster than that before dementia diagnosis, although the difference between these slopes was not statistically significant (−0.046, 95% CI −0.134 to 0.041, p=0.289).

## Discussion

Within this community-based cohort study, we found that psychological well-being declined more rapidly in individuals who went on to develop MCI compared with those who remained cognitively intact, resulting in a lower level of well-being 2 years before MCI diagnosis. Of the six components of psychological well-being examined, lower levels of purpose in life and personal growth were observed beginning 3 and 6 years before MCI diagnosis, respectively. After an MCI diagnosis, the slopes of well-being change did not significantly differ between those who did and did not develop dementia. Together, these results indicate that lower levels of psychological well-being, specifically purpose in life and personal growth, might be predictors for further cognitive impairments. Interventions or strategies aimed at enhancing well-being should be implemented earlier, before the onset of apparent cognitive impairment, to maximise their benefits for cognitive health.

There may be bidirectional associations between well-being and cognitive function. A recent meta-analysis of eight studies reported an association between greater meaning and purpose in life and lower risk of dementia.^9^ In MAP, we previously found that purpose in life—one aspect of psychological well-being—was associated with a lower risk of MCI.^5^ Moreover, rapid cognitive decline may predict subsequent lower levels of psychological well-being.^12^ To understand the potential role of psychological well-being as a predictor of dementia risk and to inform healthcare planning for the psychological needs of people with dementing disorders, more evidence is warranted about changes in well-being along the continuum of dementing disorders, especially MCI—a transient state between normal cognition and dementia. In this study, we extended our previous research to explore the trajectories of well-being before and after the diagnosis of MCI or dementia. We found that well-being was significantly lower beginning 2 years before MCI diagnosis, whereas well-being trajectory after MCI diagnosis remained similar regardless of subsequent dementia diagnosis. These findings indicate that reduced psychological well-being even without apparent cognitive impairment may be a predictor of subsequent dementing disorders. Additionally, after MCI or dementia diagnosis, the rate of decline in psychological well-being tended to accelerate, though it did not differ significantly from pre-diagnosis levels, partly due to the limited sample size. These results also echo the importance of post-diagnostic psychological support proposed in the World Alzheimer’s Report 2022.^2^

Psychological well-being is a multifactorial construct that includes various components, such as self-acceptance, autonomy, environmental mastery, purpose in life, positive relations with others, and personal growth. Of these factors, purpose in life—that is, an individual’s sense of having personally meaningful goals and directions in life—has been extensively studied,^5 8 9^ but evidence about other components of well-being is limited. In a cross-sectional study, healthy participants had higher levels of positive relationships, autonomy, personal growth, and purpose in life compared with those diagnosed with Alzheimer’s disease, whereas no significant differences were observed for self-acceptance and environmental mastery.^13^ Within a large sample of 10 099 participants from the Health and Retirement Study, purpose in life (but not life satisfaction, optimism, mastery, or positive affect) was associated with an increased risk of dementia.^7^

In this study, we found that different well-being dimensions followed different trajectories preceding MCI diagnosis. Purpose in life and personal growth became significantly lower beginning 3 and 6 years before MCI diagnosis, respectively. However, levels of self-acceptance, environmental mastery, and positive relations with others were unchanged. One potential explanation for the discrepancies across well-being components may be differences in the level of cognitive processing required.^23^ Our findings indicate that personal growth and purpose in life may be more cognitively demanding than other components of well-being, and therefore may serve as more sensitive indicators of cognitive ageing. Moreover, we found that positive relations with others declined rapidly after MCI diagnosis. People with impaired cognitive function may be less likely to engage in social and leisure activities than they were previously,^24^ which can cause further deterioration in their relationships with friends or others. Previous studies have shown that certain interventions, such as retrospective proactive life review,^25^ promoting an adult resilience programme,^26^ and a meditation-training programme,^27^ may improve well-being. Future intervention studies are warranted to explore how improvements in well-being may enhance cognitive resilience and thus prevent or delay the onset of dementing disorders.

The mechanisms underlying the association between well-being and cognitive function are not well understood. It is possible that greater well-being and better cognitive function share protective factors. Participants with higher levels of well-being tend to have lower levels of depression, smoke less, and engage in more physical, mental and social activities, all of which have been identified as protectors against dementing disorders.^428^,^30^ However, after we further controlled for these possible factors, psychological well-being still declined faster among participants with incident MCI, which may suggest the independent predictive role of well-being for cognitive ageing. This finding echoed our previous work that a greater level of psychological well-being may exhibit better cognitive resilience, which could mitigate adverse effects of Alzheimer’s disease pathology on cognition.^11 31^ Furthermore, the associations between well-being and cognitive function may be explained by the interplay between biological and psychological factors. High well-being has been linked with lower levels of inflammatory cytokines and lower cardiovascular risk, which can in turn reduce the risk of cognitive impairments.^32^

### Strengths and limitations

The strengths of this study include the use of a longitudinal cohort study with a relatively large sample size and a long-term follow-up. Well-being and its various components were collected annually, and clinical diagnosis of MCI and dementia were made based on detailed clinical examinations. However, several limitations should be noted. First, each aspect of psychological well-being was derived from only three items, which may introduce measurement errors. Future studies with data on psychological well-being from more comprehensive measurement scales are warranted to clarify the trajectories of well-being along the dementia continuum.^33^ Second, statistical power may be limited because of the relatively few observations available for some time points apart from year 0. MAP is an ongoing study, and this limitation would be addressed in our future work. Third, the study population consisted of volunteers from communities who had a high level of education, which may introduce selection bias because of healthy volunteer effect. Moreover, most participants are white and female, which may limit the generalisability of our findings to other populations. Finally, the effect sizes in our study were not substantial though statistically significant. This may be partly attributed to the underestimation owing to the inclusion of healthy volunteers in the study population. Future studies with larger and more diverse samples are warranted to verify our findings.

## Conclusions

This study provides evidence on the trajectories of psychological well-being and its various components along the continuum of MCI and dementia. We found that well-being became significantly lower preceding MCI diagnosis, whereas after MCI diagnosis, well-being declined in a similar pattern regardless of whether developing dementia or not. Our findings suggest that reduced psychological well-being, even in the absence of evident cognitive impairment, may serve as a predictor for impaired cognitive function in the long-term. Further research is needed to investigate whether and to what extent interventions targeting these well-being components may benefit cognitive function and help prevent the development of dementia.

## Supplementary material

10.1136/jnnp-2024-333837 online supplemental file 1

## Data Availability

Data are available upon reasonable request.
