# Supplementary material for: Psychological well-being trajectories preceding incident mild cognitive impairment and dementia
Source: J Neurol Neurosurg Psychiatry. 2024 Aug 13;96(3):e333837. doi: 10.1136/jnnp-2024-333837 (PMC11822041; doi:10.1136/jnnp-2024-333837)
Supplement: online supplemental file 1 [file jnnp-96-3-s001.docx]

**Supplementary file**

910 Cognitively intact participants at baseline (i.e., 2008)

Analysis of well-being trajectories before and after MCI: 910 participants (265 incident MCI and 645 cognitively intact ones)

265 Participants with incident MCI (89 developed to dementia)

Analysis of well-being trajectories before and after dementia among incident MCI cases: 229 participants (73 incident dementia and 156 dementia-free ones)

Excluding 36 participants without wellbeing after MCI diagnosis

**Supplementary Figure 1. Flow chart of the study population**

**Supplementary Text 1. Piecewise linear mixed-effect model**

The piecewise linear mixed-effects model used in the analyses of the trajectories of psychological well-being before and after MCI diagnosis is as follows.

$$time spline=\left\{ \begin{aligned} 0 if time \leq0 \\ time if time>0 \end{aligned} \right.$$

$$y=\beta_{0}+\beta_{1}time+\beta_{2}time spline+\beta_{3}MCI+\beta_{4}MCI*time+\beta_{5}age+\beta_{6}sex+\beta_{7}education+Z\gamma+ɛ$$

- y is the psychological well-being or its components
- time is aligned at the year of diagnosis for MCI ones and at the end of follow-up for cognitively intact ones
- time spline is created based on time and break point (i.e., year 0, the diagnosis year for MCI)
- MCI is a binary variable of mild cognitive impairment (Yes vs. No)
- age is the participant’s age at year 0, i.e., MCI diagnosis age for those with MCI and age at the end of follow-up for those cognitively intact
- Z is the known matrix of random effects
- γ is the unknown vector of random-effects parameters
- ε is the unobserved vector of random errors.

The piecewise linear mixed-effects model used in the analyses of the trajectories of psychological well-being before and after dementia diagnosis is as follows.

$$time spline=\left\{ \begin{aligned} 0 if time \leq0 \\ time if time>0 \end{aligned} \right.$$

$$y=\beta_{0}+\beta_{1}time+\beta_{2}time spline+\beta_{3}dementia+\beta_{4}dementia*time+\beta_{5}age+\beta_{6}sex+\beta_{7}education+Z\gamma+ɛ$$

- y is the psychological well-being or its components
- time is aligned at the year of diagnosis for dementia ones and at the end of follow-up for dementia-free ones
- time spline is created based on time and break point (i.e., year 0, the diagnosis year for dementia)
- dementia is a binary variable of dementia onset (Yes vs. No)
- age is the participant’s age at year 0, i.e., dementia diagnosis age for those with dementia and age at the end of follow-up for those who were dementia-free
- Z is the known matrix of random effects
- γ is the unknown vector of random-effects parameters
- ε is the unobserved vector of random errors.

**Supplementary Table 1. Psychological well-being items**

| **Well-being** | **Item no.** | **Item** |
| --- | --- | --- |
| Positive relations with others | 1 | Maintaining close relationships has been difficult and frustrating for me. |
|  | 2 | People would describe me as a giving person, willing to share my time with others. |
|  | 3 | I have not experienced many warm and trusting relationships with others. |
| Self-acceptance | 4 | I like most parts of my personality. |
|  | 5 | When I look at the story of my life, I am pleased with how things have turned out so far. |
|  | 6 | In many ways I feel disappointed about my achievements in life. |
| Autonomy | 7 | I tend to be influenced by people with strong opinions. |
|  | 8 | I have confidence in my own opinions, even if they are different from those of others. |
|  | 9 | I judge myself by what I think is important, not by the values of others. |
| Personal growth | 10 | For me, life has been a continuous process of learning, changing, and growing. |
|  | 11 | I think it is important to have new experiences that challenge how I think about myself and the world. |
|  | 12 | I gave up trying to make big improvements or changes in my life a long time ago. |
| Environmental mastery | 13 | The demands of everyday life often get me down. |
|  | 14 | In general, I feel I am in charge of the situation in which I live. |
|  | 15 | I am good at managing the responsibilities of daily life. |
| Purpose in life | 16 | Some people wander aimlessly through life, but I am not one of them. |
|  | 17 | I live life one day at a time and don't really think about the future. |
|  | 18 | I sometimes feel as if I've done all there is to do in life. |

**Supplementary Table 2. Multi-adjusted β coefficient (95% CI) of psychological well-being change before and after mild cognitive impairment diagnosis**

| **Model terms** | **β coefficient (95% CI)** | ***P* value** |
| --- | --- | --- |
| Slope before MCI diagnosis for cognitively intact subjects | -0.012 (-0.018, -0.005) | <0.00 |
| Slope before MCI diagnosis for incident MCI subjects | -0.024 (-0.034, -0.013) | <0.001 |
| Difference in slope before MCI diagnosis | -0.012 (-0.024, 0.000) | 0.058 |
| Slope after MCI diagnosis for incident MCI subjects | -0.043 (-0.058, -0.028) | <0.001 |
| Difference in slope before and after MCI diagnosis | -0.019 (-0.040, 0.002) | 0.076 |

Model was adjusted for age at time 0, sex, education, physical activity, body mass index, any of vascular disease risk factors, any of vascular diseases, depression syndrome, apolipoprotein E ε4 carrier, social activity, social network, and loneliness.

**
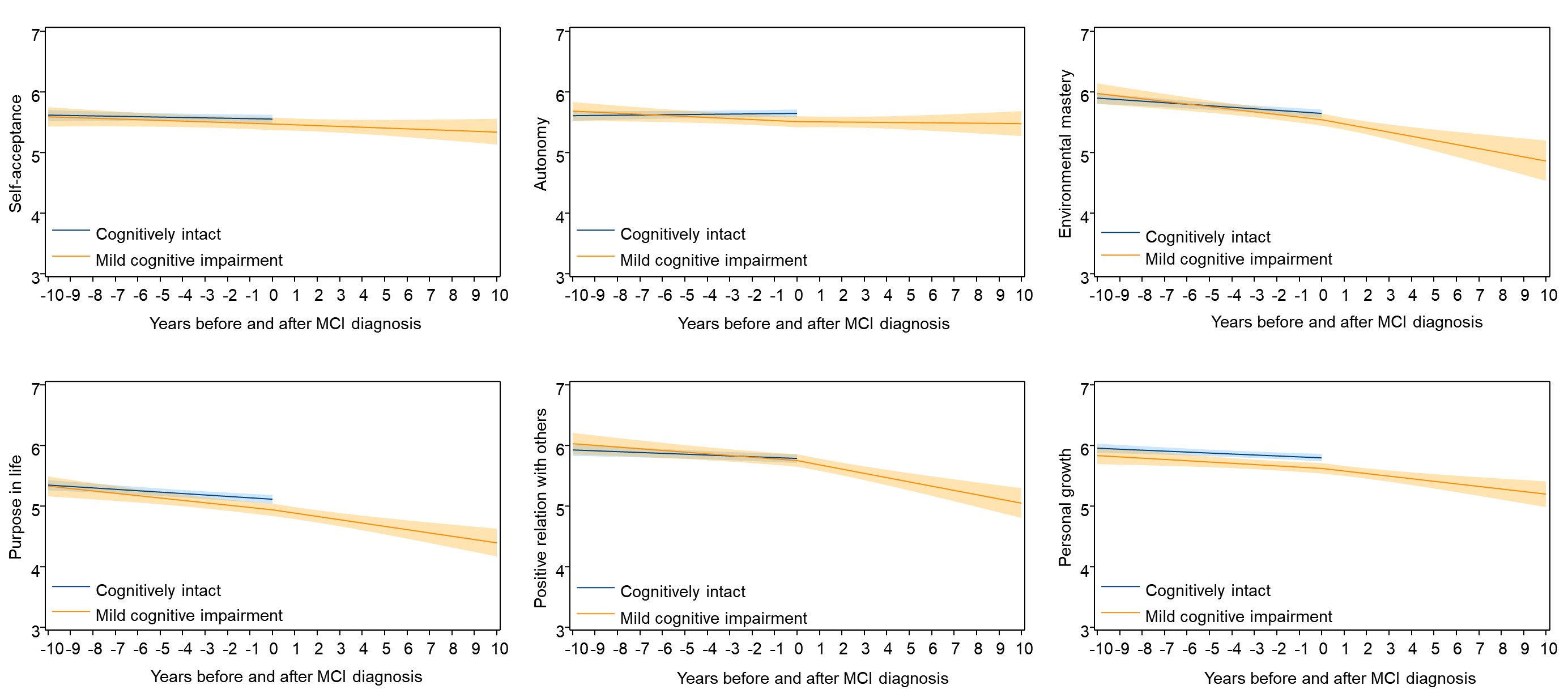
**

**Supplementary Figure 2. Trajectories of psychological well-being components before and after mild cognitive impairment diagnosis.**

Model was adjusted for age at time 0, sex, and education.

Year 0 indicates the year of MCI diagnosis or the year corresponding to the end of follow-up (for participants who remained cognitively intact).

**Supplementary Table 3. β coefficient (95% CI) of six components of psychological well-being change before and after mild cognitive impairment diagnosis.**

| **Model terms** | **Basic-adjusted β coefficient (95% CI)** | ***P* value** |  | **Multi-adjusted β coefficient (95% CI)** | ***P* value** |
| --- | --- | --- | --- | --- | --- |
| **Self-acceptance** |  |  |  |  |  |
| Slope before MCI diagnosis for cognitively intact subjects | -0.006 (-0.015, 0.002) | 0.156 |  | -0.005 (-0.014, 0.005) | 0.323 |
| Slope before MCI diagnosis for incident MCI subjects | -0.012 (-0.028, 0.005) | 0.171 |  | -0.008 (-0.024, 0.009) | 0.353 |
| Difference in slope before MCI diagnosis | -0.005 (-0.024, 0.014) | 0.588 |  | -0.003 (-0.022, 0.016) | 0.759 |
| Slope after MCI diagnosis for incident MCI subjects | -0.013 (-0.034, 0.009) | 0.237 |  | -0.011 (-0.033, 0.011) | 0.347 |
| Difference in slope before and after MCI diagnosis | -0.001 (-0.033, 0.03) | 0.928 |  | -0.003 (-0.035, 0.029) | 0.867 |
| **Autonomy** |  |  |  |  |  |
| Slope before MCI diagnosis for cognitively intact subjects | 0.004 (-0.004, 0.012) | 0.312 |  | 0.005 (-0.004, 0.014) | 0.276 |
| Slope before MCI diagnosis for incident MCI subjects | -0.017 (-0.033, -0.001) | 0.037 |  | -0.007 (-0.024, 0.009) | 0.390 |
| Difference in slope before MCI diagnosis | **-0.021 (-0.04, -0.003)** | **0.020** |  | -0.012 (-0.031, 0.007) | 0.202 |
| Slope after MCI diagnosis for incident MCI subjects | -0.003 (-0.025, 0.019) | 0.780 |  | -0.008 (-0.031, 0.016) | 0.519 |
| Difference in slope before and after MCI diagnosis | 0.014 (-0.017, 0.045) | 0.367 |  | 0.000 (-0.032, 0.032) | 0.981 |
| **Environmental mastery** |  |  |  |  |  |
| Slope before MCI diagnosis for cognitively intact subjects | -0.025 (-0.035, -0.015) | <0.001 |  | -0.026 (-0.038, -0.015) | <0.001 |
| Slope before MCI diagnosis for incident MCI subjects | -0.043 (-0.062, -0.024) | <0.001 |  | -0.040 (-0.059, -0.020) | <0.001 |
| Difference in slope before MCI diagnosis | -0.018 (-0.04, 0.004) | 0.109 |  | -0.014 (-0.036, 0.009) | 0.238 |
| Slope after MCI diagnosis for incident MCI subjects | -0.068 (-0.102, -0.033) | 0.000 |  | -0.058 (-0.092, -0.024) | 0.001 |
| Difference in slope before and after MCI diagnosis | -0.025 (-0.068, 0.018) | 0.256 |  | -0.018 (-0.061, 0.025) | 0.416 |
| **Purpose in life** |  |  |  |  |  |
| Slope before MCI diagnosis for cognitively intact subjects | -0.023 (-0.033, -0.014) | <0.001 |  | -0.021 (-0.032, -0.010) | 0.000 |
| Slope before MCI diagnosis for incident MCI subjects | -0.039 (-0.057, -0.021) | <0.001 |  | -0.038 (-0.057, -0.019) | 0.000 |
| Difference in slope before MCI diagnosis | -0.015 (-0.036, 0.005) | 0.140 |  | -0.017 (-0.039, 0.005) | 0.137 |
| Slope after MCI diagnosis for incident MCI subjects | -0.054 (-0.077, -0.031) | <0.001 |  | -0.059 (-0.083, -0.035) | <0.001 |
| Difference in slope before and after MCI diagnosis | -0.015 (-0.049, 0.018) | 0.370 |  | -0.021 (-0.057, 0.014) | 0.244 |
| **Positive relation with others** |  |  |  |  |  |
| Slope before MCI diagnosis for cognitively intact subjects | -0.014 (-0.023, -0.005) | 0.002 |  | -0.014 (-0.024, -0.003) | 0.009 |
| Slope before MCI diagnosis for incident MCI subjects | -0.028 (-0.045, -0.01) | 0.002 |  | -0.025 (-0.043, -0.007) | 0.008 |
| Difference in slope before MCI diagnosis | -0.014 (-0.033, 0.006) | 0.170 |  | -0.011 (-0.032, 0.010) | 0.296 |
| Slope after MCI diagnosis for incident MCI subjects | -0.07 (-0.095, -0.046) | <0.001 |  | -0.072 (-0.097, -0.046) | <0.001 |
| Difference in slope before and after MCI diagnosis | **-0.042 (-0.075, -0.009)** | **0.012** |  | **-0.047 (-0.082, -0.012)** | **0.009** |
| **Personal growth** |  |  |  |  |  |
| Slope before MCI diagnosis for cognitively intact subjects | -0.015 (-0.023, -0.007) | 0.000 |  | -0.012 (-0.021, -0.003) | 0.012 |
| Slope before MCI diagnosis for incident MCI subjects | -0.021 (-0.037, -0.005) | 0.008 |  | -0.018 (-0.034, -0.001) | 0.033 |
| Difference in slope before MCI diagnosis | -0.006 (-0.024, 0.012) | 0.501 |  | -0.006 (-0.025, 0.013) | 0.541 |
| Slope after MCI diagnosis for incident MCI subjects | -0.043 (-0.065, -0.021) | 0.000 |  | -0.049 (-0.071, -0.027) | <0.001 |
| Difference in slope before and after MCI diagnosis | -0.021 (-0.052, 0.009) | 0.173 |  | -0.031 (-0.063, 0.000) | 0.051 |

Basic-adjusted model was adjusted for age at time 0, sex, and education. Multi-adjusted model was adjusted for age at time 0, sex, education, physical activity, body mass index, any of vascular disease risk factors, any of vascular diseases, depression syndrome, apolipoprotein E ε4 carrier, social activity, social network, and loneliness.

**Supplementary Table 4. Differences in psychological well-being between dementia cases and non-dementia ones in the 5 years before dementia diagnosis among participants with incident mild cognitive impairment.**

| Year | No. of non-dementia | No. of dementia | Difference in mean (95% CI) | *P*-value |
| --- | --- | --- | --- | --- |
| -5 | 51 | 16 | -0.091 (-0.315, 0.134) | 0.397 |
| -4 | 67 | 21 | -0.094 (-0.291, 0.103) | 0.387 |
| -3 | 92 | 33 | -0.097 (-0.276, 0.081) | 0.387 |
| -2 | 110 | 47 | -0.101 (-0.272, 0.071) | 0.344 |
| -1 | 130 | 66 | -0.104 (-0.281, 0.074) | 0.344 |
| 0 | 156 | 73 | -0.107 (-0.302, 0.088) | 0.387 |

Difference in mean was calculated as the mean of well-being in participants with dementia minus that in those cognitively intact. Negative value means that well-being was poorer in participants with dementia. Model was adjusted for age at time 0, sex, and education.

Year 0 indicates the year of dementia diagnosis or the year corresponding to the end of follow-up (for participants who were dementia-free).

To account for the multiple testing, *P*-values and confidence intervals (CIs) in the table were calculated using a simulation-based approach combined with a step-down fashion.
